# 

**Published:** 1891-04

**Authors:** 


### Editorial, Etc.

### MISSOURI STATE DENTAL ASSOCIATION.

### PARTIAL PROGRAMME.

LOUISIANA, MO., JULY 7, 8, 9, 10, 1891.

PAPERS.

1. Dr. J. F. McWilliams, Mexico—"President's Address to State Association." Discussion opened by Dr. J. B. Newby, St. Louis.

2. Dr. J. D. Patterson, Kansas City—"Medication in the Treatment of Pulp Canals." Discussion opened by Dr. Theo. Stanley, Kansas City.

3. Dr. B. Q. Stevens, Hannibal—"The Preparation of the Mouth, and Plate Work." Discussion opened by Dr. Wm. N. Morrison, St. Louis.

4. Dr. Frank Slater, Rich Hill—"The Devitalization and Removal of Tooth Pulps." Discussion opened by Dr. J. E. Crozier, Lee's Summit.

5. Dr. J. D. McMillen, Kansas City—"Crown and Bridge Work—Use and Abuse of Same." Discussion opened by Dr. T. M. Nicholson, Fayette.

6. Dr. A. H. Fuller, St. Louis—"Preparation of Cavities." Discussion opened by Dr. H. S. Lowry, Kansas City.

7. Dr. Wm. H. Eames, St. Louis—"Review of Dr. Miller's Work, with Personal Observations." Discussion opened by Dr.————

8. Dr. Geo. L. Shepard, Sedalia—"Dental Fees." Discussion opened by Dr. L. A. Young, Neosho.

9. Dr. J. M. Austin, St. Joseph.—"Report on New Appliances." Dr. A. J. Prosser, St. Louis—"Report on Clinics."

10. Dr. I. D. Pearce, Kansas City—"General Anæsthetics in Relation to Oral Surgery." Discussion opened by Dr. L. E. Day, Nevada.

11. Dr. E. E. Shattuck, Kansas City—"Regulating Appliances." Discussion opened by Dr. W. L. Reed, Mexico.

CLINICS.

1. Dr. J. D. Patterson, Kansas City—"Pulp Canal Filling."

2. Dr. J. Stephen Coyle, St. Louis—"Open-faced Crowns."

3. Dr. J. F. Hassell, Lexington—"Soft Gold Filling."

4. Dr. E. E. Shattuck, Kansas City, will exhibit "Regulating Appliances."

5. Dr. N. W. Pence, St. Louis—"Gold Filling, Operator doing his own Malleting."

6. Dr. R. R. Vaughan, Fulton—"Copper Amalgam. Will also exhibit Bridge Work."

7. Dr. D. J. McMillen, Kansas City—"Soft Gold Filling."

8. Dr. B. Q. Stevens, Hannibal—"Amalgam Filling, to demonstrate packing and finishing. Will exhibit dies for striking up Face Teeth for Plate Work—Combination Fillings—Pulp Canal Impressions."

9. Dr. J. T. Fry, Moberly—"Compound Gold Filling."

10. Dr. J. B. Vernon, St. Louis—"Bicuspid Crown—Porcelain Face."

11. Dr. T. B. Carr, Stanberry—"Extracting."

12. Dr. E. B. Crane, California—"The Crane Vulcanizer and a new method of moulding Rubber Plates. Will also show plates made the old way."

13. Dr. W. H. Buckley, Liberty—"Small Bridge Richmond Crown Attachments."

14. Dr. Geo. A. McMillen, Alton, Ill., will show the best Foot Blow Pipe for country dentists—those who have no Gas."

15. Dr. W. M. Carter, Sedalia—"Gold Filling, using Williams' Crystalloid Gold, No. 3."

16. Dr. Wm. N. Morrison, St. Louis—"Lining Cavities with Gold and Platinum Folds, for Amalgam Fillings."

17. Dr. Wm. H. Eames, St. Louis—"New Form of Bridge Work; Teeth placed in position after Bridge is completed."

18. Dr. A. J. McDonald, Kansas City—Open Faced Bicuspids for Bridge Work."

19. Dr. A. C. Griggs, Warrensburg—"Cleft Palate Appliances."

The usual railroad rates will be given: a fare and a third on the certificate plan. Be sure to get your receipt at starting point.

Hotel rates, $1.00 and $1.50 a day. A cordial invitation is extended to all reputable dentists; come, and take part.

Dental depots will be represented. Electric motor and appliances will be exhibited. Those having new appliances should send them to Dr. J. M. Austin, St. Joseph, Mo.

WM. CONRAD,
HENRY FISHER, } *Ex. Com.*
J. W. WHIPPLE,

The Florida State Dental Association will hold its next annual meeting in Jacksonville, June 2nd to 4th.

GEO. H. PERINE, *President.*

WM. BAYLOR, *Cor. Sec'y.*

### MINNESOTA STATE DENTAL ASSOCIATION.

The Minnesota State Dental Association will hold its regular annual meeting July 8, 9 and 10th in St. Paul.

L. D. LEONARD, *Sec'y.*

### To Readers and Correspondents.

Communications intended for publication, and exchange journals and papers, should be addressed to the Editor of The American Journal of Dental Science, No. 845 N. Eutaw St. Baltimore, Md. All letters relating to the business of the Journal, or containing cash remittances, to be directed to Messrs. Snowden & Cowman, No. 9 W. Fayette Street, Baltimore, Md. Articles for publication should be received by the Editor at least two weeks previous to the first of the month on which the number in which it is designed for them to appear, is issued.

☞ The American Journal of Dental Science will contain fifty pages of reading matter in each number, making a volume of about Six Hundred pages,

EXCLUSIVE OF ADVERTISEMENTS.

